# The Signal Peptide and Chaperone UNC93B1 Both Influence TLR8 Ectodomain Intracellular Endosomal Localization

**DOI:** 10.3390/vaccines10010014

**Published:** 2021-12-23

**Authors:** Da Ao, Xueliang Liu, Sen Jiang, Yulin Xu, Wanglong Zheng, Nanhua Chen, François Meurens, Jianzhong Zhu

**Affiliations:** 1 Comparative Medicine Research Institute, Yangzhou University, Yangzhou 225009, China; 13383976630@163.com (D.A.); xueliangaaa@foxmail.com (X.L.); jiangsen8888888@163.com (S.J.); ylxu15650096726@163.com (Y.X.); 007297@yzu.edu.cn (W.Z.); hnchen@yzu.edu.cn (N.C.); 2 College of Veterinary Medicine, Yangzhou University, Yangzhou 225009, China; 3 Joint International Research Laboratory of Agriculture and Agri-Product Safety, Yangzhou 225009, China; 4 Jiangsu Co-Innovation Center for Prevention and Control of Important Animal Infectious Diseases and Zoonoses, Yangzhou 225009, China; 5 School of Pharmaceutical, Changzhou University, Changzhou 213164, China; 6 BIOEPAR, INRAE, Oniris, 44307 Nantes, France; francois.meurens@inra.fr; 7 Department of Veterinary Microbiology and Immunology, Western College of Veterinary Medicine, University of Saskatchewan, Saskatoon, SK S7N 5E2, Canada

**Keywords:** TLR8, ectodomain, intracellular localization, posttranslational modification, signal peptide, UNC93B1

## Abstract

Toll-like receptor 8 (TLR8) is a single-stranded RNA sensing receptor and is localized in the cellular compartments, where it encounters foreign or self-nucleic acids and activates innate and adaptive immune responses. However, the mechanism controlling intracellular localization TLR8 is not completely resolved. We previously revealed the intracellular localization of TLR8 ectodomain (ECD), and in this study, we investigated the mechanism of the intracellular localization. Here we found that TLR8 ECDs from different species as well as ECDs from different TLRs are all intracellularly localized, similarly to the full-length porcine TLR8. Furthermore, porcine, bovine, and human TLR8 ECDs are all localized in cell endosomes, reflecting the cellular localization of TLR8. Intriguingly, none of post-translational modifications at single sites, including glycosylation, phosphorylation, ubiquitination, acetylation, and palmitoylation alter porcine TLR8-ECD endosomal localization. Nevertheless, the signal peptide of porcine TLR8-ECD determines its endosomal localization. On the other hand, signaling regulator UNC93B1 also decides the endosomal localization of porcine, bovine, and human TLR8 ECDs. The results from this study shed light on the mechanisms of not only TLR8 intracellular localization but also the TLR immune signaling.

## 1. Introduction

Innate immunity is the first line of defense against pathogens. Pattern recognition receptors (PRRs) are utilized by innate immunity to sense dangerous signals, pathogen-associated molecular patterns (PAMPs), and damage-associated molecular patterns (DAMPs) to fight pathogens and maintain homeostasis [1,2]. Amongst the PRRs, the Toll-like receptor (TLR) family members are key components of mammalian innate immunity and are part of the primary surveillance mechanisms responding to infection [3]. Toll-like receptors (TLRs) are type I transmembrane glycoproteins, which are expressed either on the cell surface or inside the cells. Thus far, 13 types of mammalian TLRs have been found, which can recognize a variety of ligands, and the cellular localization of different TLRs are related to ligand recognition [4,5]. TLR1, TLR2, TLR4, TLR5, and TLR6 are located on the cell surface and are mostly responsible for the recognition of bacterial products. TLR3, TLR7, TLR8, and TLR9 are located in intracellular compartments and are mainly involved in the sensing of microbe’s nucleic acids. TLR activation can not only induce the secretion of inflammatory cytokines and type I interferons but also initiate adaptive immunity [6].

TLR8, TLR7, and TLR9 have conserved structure, cell localization, and activation mechanism and constitute a subfamily amongst TLRs [7]. Similar to other TLRs, TLR8 is a type I transmembrane glycoprotein with a ligand-binding large N-terminal ectodomain (ECD), a single transmembrane helical region, and a C-terminal cytoplasmic toll-interleukin-1 receptor (TIR) domain, among which, TLR8-ECD consists of 26 conserved leucine-rich repeats (LRRs) with a 30–40 amino acid long Z-loop domain between LRR14 and LRR15 [8]. TLR8 and TLR7 can recognize single-stranded RNA (ssRNA) and small molecular imidazoquinoline derivatives and other ligands [9]. Ligand binding to ECD induces TLR8 dimerization, allowing the signal adaptor protein MyD88 to be recruited by the TLR8 cytoplasmic TIR domain, ultimately triggering the activation of nuclear factor NF-κB and mediating the expression of various pro-inflammatory cytokines [10].

In TLR8 and other TLRs, ECD is responsible for the recognition of ligands and plays a key role in the ligand stimulus-response of TLRs. Our previous study on TLR8 has shown that TLR8-ECD post-translational modifications have a significant effect on TLR8 signal transduction [11]. Further, we and others found that TLR8-ECD itself is localized in the same cell area as the full-length TLR8 protein, which, together with transmembrane and cytoplasmic regions, determine TLR8 intracellular localization [12,13]. Therefore, the subcellular localization of mammalian TLR8-ECD and the influencing factors need to be investigated.

In this study, we confirmed that TLR8 ECDs from different species and even ECDs of different TLRs are all intracellularly localized. Moreover, TLR8 ECDs of the different species are all localized in cell endosomes as the full-length form. Further, we analyzed the effects of porcine TLR8-ECD signal peptides and post-translational modifications at single sites, including glycosylation, phosphorylation, ubiquitination, acetylation, and palmitoylation on the subcellular localization of TLR8-ECD. Lastly, we examined the effects of TLR transporter chaperone UNC93B1 on the subcellular localization of various TLR8-ECDs. We found that both the signal peptide and UNC93B1 play a role in the intracellular localization of TLR8-ECD.

## 2. Materials and Methods

### 2.1. Cells and Reagents

The human embryonic kidney cell line HEK293T were maintained in Dulbecco’s modified Eagle’s medium (DMEM, Hyclone Laboratories, Logan, UT, USA) supplemented with 10% fetal bovine serum (FBS) and 100 IU/mL of penicillin plus 100 μg/mL streptomycin (Hyclone Laboratories). Porcine alveolar macrophages (PAMs) cell line (3D4/21) was cultured in Roswell Park Memorial Institute 1640 (RPMI, Hyclone, Logan, UT, USA) containing 10% FBS with penicillin/streptomycin. All cells were maintained at 37 °C with 5% CO_2_ in a humidified incubator.

Restriction endonucleases *BsmB* I (#R0580L) and *Dpn* I (R0176S), T4 polynucleotide kinase (#M0201V), Phusion Hot Start High Fidelity DNA polymerase (M0203S), and T4 DNA ligase (M0203S) were all purchased from New England Biolabs (Beijing, China). 2 × MultiF Seamless Assembly Mix (RK21020) was bought from ABclonal (Wuhan, China). Blue Plus^®^ IV Protein Marker (10–180 kDa) (DM131-01), ProteinFind^®^ Anti-GFP Mouse Monoclonal Antibody (HT801-01), ProteinFind^®^ Anti-HA Mouse Monoclonal Antibody (HT301-01), ProteinFind^®^ Anti-β-Actin Mouse Monoclonal Antibody (HC201-01), ProteinFind^®^ Goat Anti-Mouse IgG (H + L), HRP Conjugate (HS201-01), EasyScript^®^ All-in-One First-Strand cDNA Synthesis SuperMix (OneStep gDNA Removal) (AE341-02) and TransDetect^®^ Double-Luciferase Reporter Assay Kit (FR201-01) were all provided by Transgen Biotech (Beijing, China). 2-(4-Amidinophenyl)-6-indolecarbamidine dihydrochloride (DAPI, C1002) was obtained from Beyotime Biotechnology (Shanghai, China).

### 2.2. Bioinformatics Analysis

SignalP 5.0 Server (http://www.cbs.dtu.dk/services/SignalP/) was used to predict the signal peptide cleavage site of porcine TLR8-ECD. NetNGlyc 1.0 Server (http://www.cbs.dtu.dk/services/NetNGlyc/) was used to predict pTLR8-ECD N-glycosylation sites. NetPhos 2.0 Server (http://www.cbs.dtu.dk/services/NetPhos-2.0/) was used to forecast pTLR8-ECD phosphorylation sites. PLMD-protein Lysine to modify the Database Server 3.0 (http://www.cbs.dtu.dk/services/NetPhos-2.0/) was used to forecast pTLR8-ECD ubiquitination sites. GPS-PAIL Online Service (http://pail.biocuckoo.org/online.php) was used to forecast pTLR8-ECD acetylation sites. Then, CSS-Palm Online Service (http://csspalm.biocuckoo.org/showResult.php) was used to forecast pTLR8-ECD palmitoylation sites. The prediction results for pTLR8-ECD were the following: signal peptide was composed of the first 19 amino acids. The N-glycosylation sites are N26, N39, N77, N85, N177, N189, N283, N348, N355, N409, N580, N678, and N741. The phosphorylation sites were Y61, T63, S335, Y338, S358, and T751. The ubiquitination sites were K233, K600, K625, and K781. The acetylation site was K748 and the palmitoylation sites were C10, C19, C260, and C792.

### 2.3. Molecular Cloning and Gene Mutations

PCR primers were designed on Benchling (https://benchling.com/) for the following genes: Porcine TLR8 (pTLR8, GenBank: EF583903.1), bovine TLR8 (bTLR8, GenBank: EF583902), human TLR8 (hTLR8, GenBank: NM_138636.5), porcine TLR3 (GenBank: NM_001097444.1), porcine TLR5 (GenBank: Nm_001348771.1), glycoprotein D (gD) of bovine herpes virus type I (BoHV-1) (GenBank: MH751901.1), porcine STING (GenBank: NM_001142838.1), human GM130 (GenBank: AF248953.1), human LAMP1 (GenBank: J04182.1), and porcine UNC93B1 (GenBank: XM_003122436.6). All the primers are available in Supplementary Table S1. The porcine TLR8, porcine TLR8-ECD, bovine TLR8-ECD, human TLR8-ECD, porcine TLR3-ECD, porcine TLR5-ECD, gD, and gD-ECD sequences were all amplified using PCR from the corresponding plasmids in our lab. For pTLR8, pTLR8-ECD, bTLR8-ECD, hTLR8-ECD, the PCR products were cloned into the *Bgl* II and *Kpn* I sites of both pEGFP-N1 and pEGFP-C1 vectors. For pTLR3-ECD and pTLR5-ECD, the PCR products were cloned into pEGFP-C1 (*Bgl* II and *Kpn* I). For gD and gD-ECD, the PCR products were cloned into pEGFP-N1 (*Bgl* II and *Kpn* I). STING, GM130, and LAMP1 were PCR amplified from cDNAs, and the PCR products were cloned into pDsRed-C1 (*Bgl* II and *Kpn* I). Porcine UNC93B1 was PCR amplified from cDNA and then cloned into *Nhe* I and *EcoR* V sites of pcDNA3.1-2HA to generate pcDNA3.1-pUNC93B1-2HA. All the PCR products were cloned into vectors either by T4 DNA ligase or by Seamless Assembly Mix.

According to the predicted results of signal peptide and post-translational modification sites of pTLR8-ECD, the mutation PCR primers were designed using the Quick-change Primer Design method (https://www.agilent.com), which are shown in Supplementary Table S2. The signal peptide deletion mutant of pTLR8-ECD was designated as pTLR8-ECD Δ-SP. The pTLR8-ECD N-glycosylation mutants generated are named: N26A, N39A, N77A, N85A, N177A, N189A, N283A, N348A, N355A, N409A, N580A, N678A, and N741A. The phosphorylation mutants were Y61A, T63A, S335A, Y338A, S358A, and T751A. The ubiquitination mutants were K233A, K600A, K625A, and K781A. The acetylation mutant was K748A, and the palmitoylation mutants were C10A, C19A, C260A, and C792A. The mutation PCR was performed using Phanta Max Super-Fidelity DNA Polymerase (Cat: P505, Vazyme Biotech, Nanjing, China) and the template TLR8-ECD plasmid. Subsequently, the PCR products were digested with *Dpn* I at 37 °C for 30 min, and the PCR products were transformed into competent XL10-gold or DMT bacteria. All the obtained mutants were DNA sequenced to ensure the desired mutations.

### 2.4. Western Blot (WB) Analysis

HEK-293T cells grown in 24-well plates (4 × 10^5^ cells/well) were transfected with various plasmids using Lipofectamine 2000 (Invitrogen, Carlsbad, CA, USA). Twenty-four hours (h) later, cells were collected and lysed in RIPA buffer (50 mM Tris pH 7.2, 150 mM NaCl, 1% sodium deoxycholate, 1% Triton X-100). Cell lysates were resolved on 6% SDS polyacrylamide gels in the presence of 2-mercaptoethanol. The protein bands on gels were transferred onto PVDF membranes, and the membranes were blocked with 5% skim milk in Tris-buffered saline, pH 7.4, with 0.1% Tween-20 (TBST) for 2 h at room temperature (RT), and then incubated with anti-GFP, anti-HA or β-Actin primary antibodies (1:1000 dilutions) for 1 h RT. After washing with TBST, the membranes were incubated with horseradish peroxidase-conjugated goat anti-mouse IgG (1:10,000 dilutions) for 1 h RT. The protein expression signals were detected with chemiluminescence substrate (Tanon, Shanghai, China) and visualized using a Western blot imaging system (Tanon, Shanghai, China).

### 2.5. Fluorescence Microscopy

HEK-293T cells were grown in 15 mm glass bottom cell culture confocal dish (Cat 801002, NEST, Wuxi, China) (3 × 10^5^ cells) for 24 h. The cells were transfected with the different plasmids in various experiments using Lipofectamine 2000 for 24 h. The pDsRed-C1-pRab5 we constructed before [14] was used to express N-terminal RFP fused pRab5 as the endosomal marker in transfected cells. The pDsRed-C1-pSTING was transfected to express N-terminal RFP fused pSTING as the ER marker. The pDsRed-C1-hGM130 was transfected to express N-terminal RFP fused hGM130 as the Golgi marker. The pDsRed-C1-hLAMP1 was transfected to express N-terminal RFP fused hLAMP1 as the lysosome marker. Cells were fixed with ice-cold 4% paraformaldehyde for 15 min and permeabilized with 0.5% TritonX-100 for 10 min at RT. Cells were then washed with PBS, counterstained with 0.5 μg/mL DAPI at 37 °C for 15 min to stain the cell nucleus. Lastly, the cells were observed under a laser-scanning confocal microscope (LSCM, Leica SP8, Solms, Germany) at the excitation wavelengths 340 nm, 488 nm, and 561 nm, respectively.

### 2.6. Promoter-Driven Luciferase Reporter Assay

HEK293T cells grown in 96-well plates (3 × 10^4^ cells/well in 100 μL media) were co-transfected using Lipofectamine 2000 with ELAM (NF-κB)-firefly luciferase (Fluc) reporter (10 ng/well) and β-actin Renilla luciferase (Rluc) reporter (0.2 ng/well), together with the different TLR8 plasmids (5 ng/well). The total DNA per well was completed to 50 ng by the addition of an empty pEGFP-C1 or pEGFP-N1 vector. About 24 h post-transfection, cells in each well were stimulated with R848 (10 μg/mL) for another 12 h. Then, the cells were harvested, lysed, and Fluc and Rluc activities were sequentially measured using the Duo-Lite Luciferase Assay System (DD1205-01, Vazyme, Nanjing, China). The results were expressed as fold induction of ELAM (NF-κB)-Fluc relative to that of vector control after Fluc normalization by corresponding Rluc.

### 2.7. Construction of UNC93B1 Gene Knockout 3D4/21 Cell Line

The CRISPR gRNA encoding DNA sequences were designed based on porcine UNC93B1 gene (Gene ID: 100522238) using the online tools in Benchling, and 3 pairs of gRNA sequences targeting porcine UNC93B1 gene exons were chosen. The synthesized gRNA encoding DNA, as shown in Supplementary Table S3 were phosphorylated by T4 polynucleotide kinase followed by ligation into the *BsmB* I linearized pLenti-CRISPRV2 vectors using T4 ligase. The recombinant plasmids were confirmed by sequencing.

HEK-293 T cells grown in 24-well plates (3 × 10^5^ cells/well in 500 μL media) were cultured for 24 h. The cells were co-transfected using Lipofectamine 2000 with the pCDNA3.1-pUNC93B1-HA plus each CRISPR-gRNA vector. After transfection for 48 h, cells of each group were collected for Western blotting detection of HA-labeled UNC93B1, and pLenti-CRISPRV2-gRNA-1 with the best knockout effect of UNC93B1 was selected for the subsequent experiments.

The lentivirus encoding UNC93B1 gRNA1 was packaged from 293T cells in a 6-well plate co-transfected with pLenti-CRISPRV2-gRNA-1, psPAX2, and pMD2.G. The pLenti-CRISPRv2 packaged virus was used as a control. 3D4/21 porcine cells grown in 6-well plates (3 × 10^5^ cells/well in 2 mL media) were infected with 1 mL gRNA or control lentiviruses in the presence of 6 μg/mL polybrene. After 72 h infection, the cells were subjected to puromycin (1.5 μg/mL) selection. After puromycin selection, surviving 3D4/21 cells from the lentivirus infected groups were further cultured. Finally, UNC93B1 KO 3D4/21 cells and vector control cells were ready for cell localization experiments.

### 2.8. Statistical Analysis

All the experiments were performed at least 3 times with similar results, and the representative experimental data were shown as average ± SD. The statistical analysis was performed using Student *t*-test provided in GraphPad Prism 5.0 (San Diego, CA, USA). A *p*-value < 0.05 was considered statistically significant.

## 3. Results

### 3.1. Expressions and Cellular Localizations of the Porcine TLR8 and Different TLR-ECD Proteins

In order to compare the effects of GFP-tag expression positions on the expression and cell localization of porcine TLR8 and TLR8-ECDs, the porcine TLR8 and the TLR8-ECDs of different species (p, b, and h) were cloned into both pEGFP-N1 and pEGFP-C1 expression vectors, in which GFP tag was fused to the C-termini and N-termini of the expressed proteins, respectively. Meanwhile, due to the cell plasma membrane localization of gD protein of BoHV-1 and the typical exocrine feature of the gD-ECD protein, gD and gD-ECD were cloned into pEGFP-N1, respectively, as controls. On the other hand, porcine TLR3-ECD and TLR5-ECD were cloned into pEGFP-C1 for examination of the expressions and cellular localizations.

**Figure 1:**
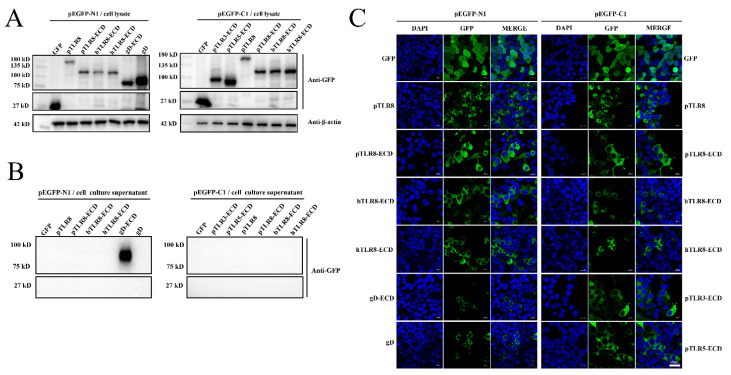
Detection of the expressions of TLR8 and different TLR-ECDs. (**A**) The plasmids of wild-type pTLR8, pTLR8-ECD, bTLR8, hTLR8, pTLR3-ECD, pTLR5-ECD, gD, gD-ECD, and vector pEGFP-C1 (0.5 µg each) were transfected into HEK-293T cells (24-well plate, 3 × 10^5^ cells/well) with Lipofectamine 2000. After 24 h, the expression of GFP fusion protein in cells was detected by WB. (**B**) At the same time, the cell culture medium was collected to detect the protein expression in the supernatants. (**C**) The plasmids including wild-type pTLR8, pTLR8-ECD, bTLR8, hTLR8, pTLR3-ECD, pTLR5-ECD, gD, gD-ECD, and vector pEGFP-C1 (0.5 µg each) were transfected into HEK-293T cells (3 × 10^5^ cells/well). After staining with DAPI, the cells were examined under a confocal microscope. The scale bar is 40 μM, and the same are for the follow-up images.

Western blotting results showed that pTLR8, pTLR8-ECD, hTLR8-ECD, bTLR8-ECD, pTLR3-ECD, pTLR5-ECD, and gD could be detected in cells after transfection (Figure 1A), but not in the supernatants of the same cells (Figure 1B). The cellular expressions of pTLR8, pTLR8-ECD, bTLR8-ECD, and hTLR8-ECD were not affected by the GFP tag positions since both N, C-terminal GFP fused pTLR8, and various TLR8-ECDs gave the same results (Figure 1A,B). In contrast, most of the gD-ECD protein was detected in the cell supernatant in spite of some expression in cells as expected (Figure 1A,B). Correspondingly, the confocal microscopy results showed that pTLR8 and all TLR-ECD proteins, including pTLR8-ECD, bTLR8-ECD, hTLR8-ECD, pTLR3-ECD, and pTLR5-ECD were all localized in the cytoplasm of the transfected cells (Figure 1C). Again, the cytoplasmic expressions of pTLR8 and various TLR8-ECDs were not affected by the GFP tag positions (Figure 1C). While gD protein was expressed on the cell plasma membrane, gD-ECD protein was mainly secreted outside the cells, and only a residual amount was still present in the cells (Figure 1C).

The above results demonstrated that, as a control, the full-length BoHV-1 gD was localized on the cell membrane, whereas its ECD was secreted out of the cells. However, different from gD-ECD, porcine, bovine, and human TLR8-ECDs all exist in the cytoplasm but are not secreted outside the cells. Additionally, as another intracellular TLR, TLR3 has its ECD expressed and localized inside cells as well. Intriguingly, TLR5, which is the cell surface TLR, has its ECD also expressed and localized inside cells as other TLR ECDs. Therefore, it appears that the cytoplasmic localization of TLR-ECD is a general phenotype for all the TLRs, and it is worthy of exploring the underlying mechanism of TLR-ECD cellular localization.

### 3.2. ER and Endosomal Localization of Porcine, Bovine, and Human TLR8-ECDs

**Figure 2:**
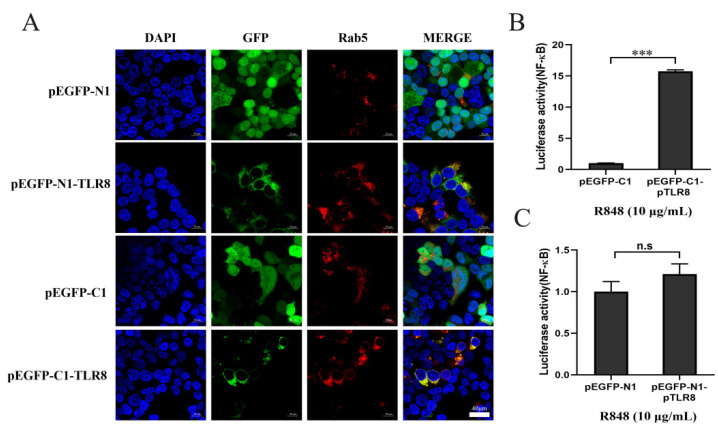
Effect of GFP label location on pTLR8 signaling activity. (**A**) The pDsRed-C1-pRab5 (0.5 µg) and pEGFP-N1, pEGFP-C1, pEGFP-N1-pTLR8, or pEGFP-C1-pTLR8 plasmids (0.5 µg each) were used to co-transfected into HEK-293T cells (3 × 10^5^ cells/well). After DAPI staining, the cells were observed using a confocal microscope. (**B**,**C**) The pEGFP-N1, pEGFP-C1, pEGFP-N1-pTLR8, or pEGFP-C1-pTLR8 (5 ng each) were co-transfected together with ElAM Fluc (10 ng) and RLuc (0.2 ng) into HEK-293T cells in 96-well plate. After 24 h, R848 (10 μg/mL) was applied for 12 h of stimulation. *** *p* < 0.001 and n.s denotes no significant difference.

In addition to the effects of GFP position on TLR8 and ECD proteins, we were also curious to know the effect of GFP tag position on TLR8 activity. Indeed, both N and C-terminal GFP tagged pTLR8 were co-localized with Rab5, an endosomal marker, indicating the endosomal localizations, whereas GFP from pEGFP-N1 and pEGFP-C1 vectors were both distributed in the whole cells (Figure 2A). Then the N-GFP and C-GFP tagged pTLR8 were tested for their signaling activities by NF-κB promoter dual luciferase assay. The results showed that after stimulation with R848, the N-GFP tagged pTLR8 from pEGFP-C1-pTLR8 (Figure 2B) but not the C-GFP tagged pTLR8 from pEGFP-N1-pTLR8 (Figure 2C) responded to R848 stimulation to mediate the downstream NF-κB activation. These results suggested that although GFP position did not change the TLR8 cellular localization, the C-terminal GFP hindered the TLR8 signal transduction and activation. Considering the physiological relevance of cellular localization of TLR within the signaling pathway, the pEGFP-C1 vector was used to express different TLR8-ECDs for the subsequent experiments.

**Figure 3:**
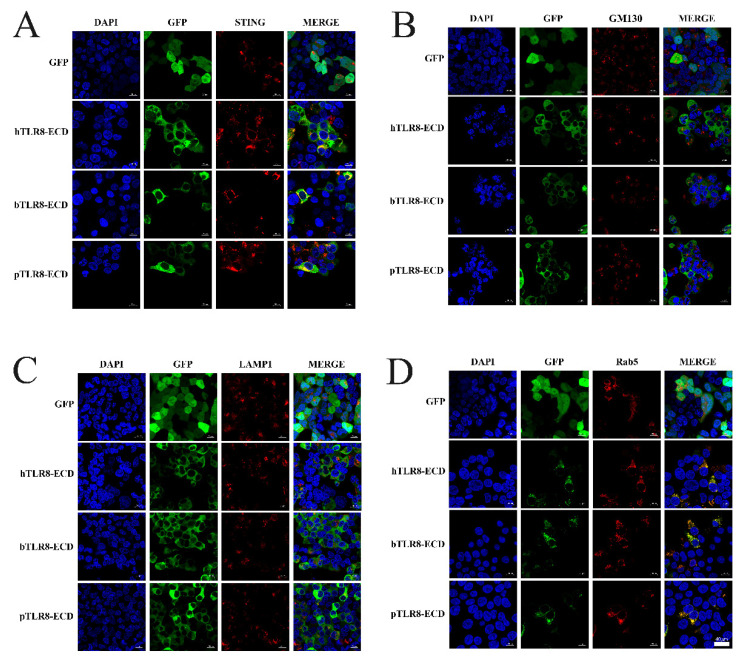
ER and endosomal localizations of porcine, bovine and human TLR8-ECDs. The pEGFP-C1 plasmids encoding hTLR8-ECD, bTLR8-ECD, or pTLR8-ECD (0.5 µg each) were each used to co-transfect HEK-293T cells (3 × 10^5^ cells/well) with pDsRed-C1-pSTING (0.5 µg) (**A**), pDsRed-C1- hGM130 (0.5 µg) (**B**), pDsRed-C1-hLAMP1 (0.5 µg) (**C**), or pDsRed-C1-pRab5 (0.5 µg) (**D**). After DAPI staining, the cells were observed using confocal microscope.

First, the pTLR8-ECD, bTLR8-ECD and hTLR8-ECD pEGFP-C1 recombinant plasmids were each co-transfected with pDsRed-C1-pSTING, which expresses ER marker protein STING. In transfected cells, the N-GFP tagged pTLR8-ECD, bTLR8-ECD, and hTLR8-ECD did co-localize with STING, whereas the GFP control from pEGFP-C1 vector did not (Figure 3A), indicating the ER localization of these TLR8 ECD proteins. Second, pTLR8-ECD, bTLR8-ECD, and hTLR8-ECD expressing pEGFP-C1 were each used to co-transfect cells with pDsRed-C1-hGM130, which expresses GM130 protein, a Golgi apparatus marker protein. In transfected cells, however, no co-localization between each of three N-GFP tagged TLR8-ECDs and GM130 was observed (Figure 3B). Third, pTLR8-ECD, bTLR8-ECD, and hTLR8-ECD expressing the pEGFP-C1 were each used to co-transfect cells with pDsRed-C1-hLAMP1, which expresses LAMP1 protein, a lysosome location protein. Again, no co-localization was observed between each GFP-tagged TLR8-ECDs and LAMP1 (Figure 3C). Finally, pTLR8-ECD, bTLR8-ECD, and hTLR8-ECD expressing pEGFP-C1 were each used to co-transfect cells with pDsRed-C1-pRab5, which expresses Rab5 protein, an endosome marker protein. The results showed that, although the control GFP from pEGFP-C1 vector did not co-localize with Rab5, pTLR8-ECD, bTLR8-ECD, and hTLR8-ECD did co-localize apparently all with Rab5 (Figure 3D), clearly demonstrating the endosomal localization of these TLR8-ECD proteins.

### 3.3. The Single Site Post-Translational Modifications Do Not Affect the Endosomal Localization of pTLR8-ECD

**Figure 4:**
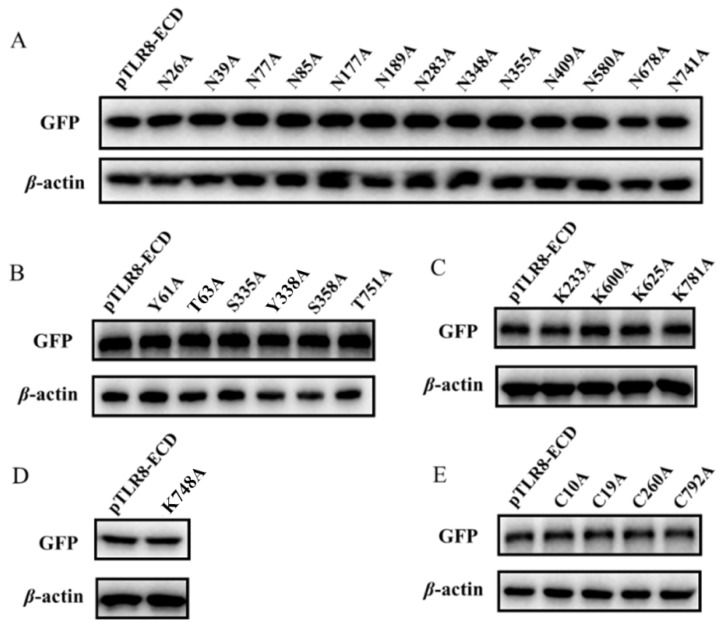
The expressions of various post-translational modifications mutants. The mutants of N-glycosylation modification sites (**A**), phosphorylated modification sites (**B**), ubiquitination modification sites (**C**), acetylation modification sites (**D**), and palmitoylation modification sites (**E**) (0.5 μg each) were transfected into HEK-293T cells (24-well plate, 3 × 10^5^ cells/well) using Lipofectamine 2000. After 24 h, the protein expression was detected by WB using the anti-GFP antibody.

The 13 predicted N-glycoylation modification sites in pTLR8-ECD were individually point mutated and mutants (N26A, N39A, N77A, N85A, N177A, N189A, N283A, N348A, N355A, N409A, N580A, N678A, and N741A) were singly tested for their expressions and cellular endosomal localization. Western blotting results showed that the above 13 N-glycosylation site mutants were all expressed with the same sizes as the wild-type pTLR8-ECD (Figure 4A). Confocal laser microscopy showed that all 13 mutants were co-localized with endosomal marker Rab5, similar to pTLR8-ECD (Supplementary Figure S1), indicating no effect of the single site N-glycosylation modifications on pTLR8-ECD localization on cell endosomes. Similarly, the pTLR8-ECD mutants of 6 phosphorylation sites (Y61A, T63A, S335A, Y338A, S358A, and T751A), 4 ubiquitination sites (K233A, K600A, K625A, and K781A), 1 acetylation site (K748A), and 4 palmitoylation sites (C10A, C19A, C260A, and C792A) were all examined for their expressions and cellular localizations. The Western blotting results showed that all these pTLR8-ECD mutants were expressed with the same sizes as the wild-type pTLR8-ECD (Figure 4B–E). However, none of the mutants exhibited an obviously altered endosomal localization using confocal microscopy (Supplementary Figures S2–S5). Taken together, these results suggested that the single site post-translational modifications do not affect the TLR8-ECD subcellular localization.

### 3.4. The Signal Peptide and UNC93B1 Contribute to the Subcellular Localizations of TLR8-ECDs

**Figure 5:**
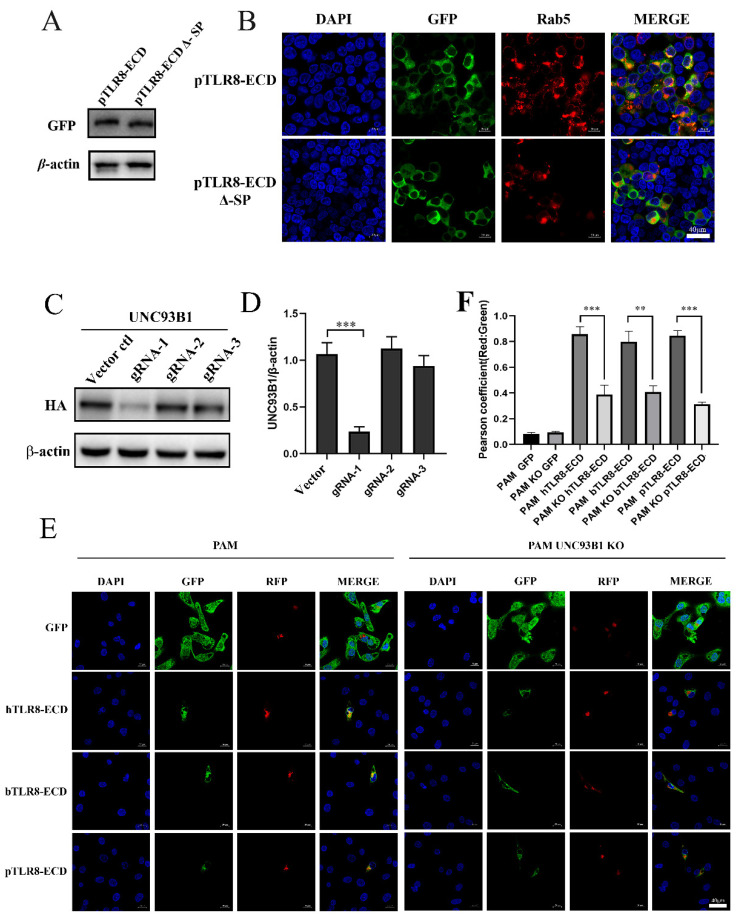
The signal peptide and UNC93B1 contribute to the subcellular localization of TLR8-ECDs. (**A**) The signal peptide deletion mutant and wild-type pTLR8-ECD plasmids (0.5 µg each) were used to transfect HEK-293T cells (24-well plate, 3 × 10^5^ cells/well) using Lipofectamine 2000. After 24 h, the protein expression was detected by WB. (**B**) HEK-293T cells (3 × 10^5^ cells/well) were co-transfected with the signal peptide deletion mutant and the wild-type pTLR8-ECD (0.5 µg each) together with pDsRed-C1-pRab5 (0.5 µg). After DAPI staining, the cells were visualized using a confocal microscope. (**C**) HEK-293T cells (24-well plate, 3 × 10^5^ cells/well) were transfected with pUNC93B1-2HA plasmid (0.5 μg) and various gRNA (1 µg each) or pLenti-CRIPRv2 vector control using Lipofectamine 2000. After 48 h, the protein expression was detected by WB with anti-HA antibody. (**D**) Gray value analysis of the HA protein bands after actin normalization in (**C**), *** *p* < 0.001. (**E**) The pDsRed-C1-pRab5 (0.5 µg) was co-transfected with pEGFP-C1, pEGFP-C1-hTLR8-ECD, pEGFP-C1-bTLR8-ECD, or pEGFP-C1-pTLR8-ECD plasmid (0.5 g each) into control PAM 3D4/21 cells or UNC93B1 KO 3D4/21 cells (2.5 × 10^5^ cells/well). After 48 h, the DAPI stained cells were visualized using the confocal microscope, with the representative images presented. (**F**) The co-localizations of various EGFP-TLR8-ECDs with DsRed-Rab5 in multiple vision fields were analyzed using Image J, and the pearson coefficient values from 10 positive cells were graphed. The value of Pearson coefficient reflects the level of co-localization, with 1 representing 100% co-localization. ** *p* < 0.01 and *** *p* < 0.001.

The predicted signal peptide sequence (amino acids 1–19) of pTLR8-ECD was deleted, and deletion mutant pTLR8-ECD Δ-SP was examined for its expression and cellular localization. Western blotting results showed that the pTLR8-ECD Δ-SP was expressed slightly smaller than wild-type pTLR8-ECD (Figure 5A). Confocal microscopy showed that whereas the wild-type pTLR8-ECD did co-localize with endosomal marker Rab5, pTLR8-ECD Δ-SP did not co-localize with Rab5 (Figure 5B). These results suggested that signal peptides in pTLR8-ECD could guide its localization on endosomes.

UNC93B1 was reported to assist the nucleic acid recognizing TLRs in transportation to endosomes [15]. In order to verify the role of signaling regulator UNC93B1 in the TLR8-ECD cellular localizations, CRISPR-gRNAs were designed to knockout the UNC93B1 in porcine alveolar macrophages (3D4/21 cells). Three pairs of gRNAs were first tested for their knockout efficiency over the ectopic UNC93B1 and the gRNA-1 was shown to be the most effective (Figure 5C,D). Based on gRNA1, the UNC93B1 KO 3D4/21 and the control cells were prepared, and both cell lines were co-transfected with pDsRed-C1-pRab5 together with the porcine, bovine or human pTLR8-ECD. In control PAM 3D4/21 cells, the three TLR8-ECDs did co-localize with the endosomal marker Rab5 (left panel, Figure 5E,F). However, in UNC93B1 KO 3D4/21 cells, no co-localizations were observed between each TLR8-ECDs and Rab5 (right panel, Figure 5E,F). These results suggested that UNC93B1 could facilitate the transport of the TLR8-ECDs of different species into endosomes in some way.

## 4. Discussion

Over the past few decades, the innate immune system has been intensively studied, especially in terms of the mechanisms developed by the TLRs to recognize pathogens [16,17]. However, the immunology mechanisms of TLRs are not totally resolved, especially in domestic animals as well as wildlife species. TLR8, TLR7, and TLR9 are all located in the endosomes and recognize microbial nucleic acids in the endosomal lumen [7,18]. A large variety of regulatory mechanisms have been identified to limit the recognition and response of these nucleic acid receptors to cellular own DNA or RNA, and the alteration of these mechanisms can lead to autoimmune or inflammatory diseases [19]. TLR intracellular transport and localization have become some of the main ways for the host to distinguish self- and non-self and to induce specific and different cell signals [4]. Studies have shown that the subcellular localization of human TLR8 is closely related to its transmembrane region [20]. During activation, TLR8 must undergo multi-step transport from the endoplasmic reticulum to endosomes via transporter UNC93B1 and maturation in an endoplasmic/lysosomal low-pH acidic environment [20]. Our previous studies on cell localization of bovine TLR8 showed that multiple regions of TLR8, including the cytoplasmic TIR region, ECD, and a transmembrane region, were all involved in the intracellular localization of TLR8, and bovine TLR8-ECD was present in the cytoplasm when expressed alone [12]. In this study, we found that the ECDs of porcine TLR8, TLR3, and TLR5 are all localized in the cytoplasm, and TLR8-ECDs from different species were also localized in the cytoplasm. These findings suggest that intracytoplasmic localization of TLR-ECD is universally similar and may be closely related to TLR signaling function. Therefore, it is meaningful to explore further the TLR8-ECD subcellular localization as well as the responsible regulation and the influencing factors.

In order to explore the subcellular localizations of porcine, bovine, and human TLR8-ECD proteins, the co-localizations of TLR8-ECDs with various organelle marker proteins including STING (ER), GM130 (Golgi), Rab5 (Endosome), and LAMP1 (Lysosome) were analyzed. It turned out that these TLR8-ECD proteins are all similarly located in the ER and endosomes, despite some species specificity of TLR8-ECD that we recently revealed [14]. The significance of TLR8-ECD localization in cell endosomes is not clear. Probably, it is a secure mechanism for TLR8 and other endosomal TLRs during evolution. The ECD of endosome localization confers the full-length TLR8 intrinsic feature to be oriented for cell endosomes. In such a way, the correct cellular endosome localization of TLR8 is ensured, thus that the appropriate cell signaling of TLR8 would be achieved.

Post-translational modifications (PTMs) of a protein are involved in the regulation of its conformation, activity, stability, and subcellular localization, playing an important role in its functions [21,22,23]. Despite the fact that that the TLR8 (hTLR8) crystal structure has been dissected by several studies [10,24,25], which significantly advanced understanding of TLR8 biology, the exact sites for post-translational modifications (PTMs) in TLR8 ECD are still unknown. In order to further explore the influencing factors of porcine TLR8-ECD endosomal localization, we examined the effects of several common predicted protein modifications, including N-glycosylation [26], phosphorylation [27], ubiquitination [28], acetylation [29], palmitoylation [30], on its endosomal localization. The results showed that all the point mutations in these PTM sites did not affect the localization of pTLR8-ECD in endosomes. It is possible that a single PTM has a limited effect on pTLR8-ECD localization, but multiple and combined PTMs may alter pTLR8-ECD cellular localization. In contrast, single point mutations of TLR8-ECD play an important role in TLR8-ECD recognition of agonists and subsequent TLR8 signaling, as we reported previously [11,14]. Whether the combined PTMs alter TLR8-ECD cellular localization and all the PTM sites are able to affect the pTLR8 signaling would need to be explored.

The signal peptide hypothesis was proposed in the early 1970s [31]. It is the N-terminal amino acid sequence used to guide protein transmembrane localization amongst the newly synthesized polypeptide chains [31,32]. The signal peptide has been widely used in the expression of eukaryotic and prokaryotic proteins. We wondered whether it had any influence on the intracellular localization of pTLR8-ECD. The results showed that the absence of pTLR8-ECD signal peptide hindered its location on the endosomes, indicating that signal peptide plays an important role in the localization of pTLR8-ECD on the endosomes.

It is important to note that TLRs (TLR3, TLR7, TLR8, and TLR9), which recognize nucleic acids, are all located in endosomes. Endosomal localization limits the recognition of self-derived nucleic acids released by the cells to avoid the occurrence of autoimmunity. The multisite membrane protein UNC93B1 is essential for intracellular localization and signal transduction of TLR via interacting with TLR3, TLR7, TLR8, and TLR9 to promote intracellular transport [33]. Our results also suggested the important role of UNC93B1 in the intracellular localization of TLR8-ECDs of different species.

## 5. Conclusions

In this study, we discovered that the TLR8 ECDs and other TLR ECDs are localized in cell endosomes as full length TLR8. Further, the single site post-translational modifications (PTMs) do not affect ECD endosomal localization, whereas the signal peptide and signaling regulator UNC93B1 determine the ECD endosomal localization. The findings help understand not only the correct TLR8 intracellular localization but also the appropriate TLR8 immune signaling.

## Data Availability

The data are all available in the paper and online.

## Supplementary Materials

The following supporting information can be downloaded at: https://www.mdpi.com/article/10.3390/vaccines10010014/s1, Figure S1: The effect of N-glycosylation modification on the subcellular localization of pTLR8-ECD; Figure S2: The effect of phosphorylated modification on the subcellular localization of pTLR8-ECD; Figure S3: The effect of ubiquitination modification on the subcellular localization of pTLR8-ECD; Figure S4: The effect of acetylation modification on the subcellular localization of pTLR8-ECD; Figure S5: The effect of palmitoylation modification on the subcellular localization of pTLR8-ECD; Figure S6: The raw data of Western blotting in this study; Table S1: Cloning PCR Primers used in this study; Table S2: Mutation PCR primers used for pTLR8-ECD mutants; Table S3: Specific guide RNA coding sequences for porcine UNC93B1 CRISPR knockout.
