# Supplementary material for: The Signal Peptide and Chaperone UNC93B1 Both Influence TLR8 Ectodomain Intracellular Endosomal Localization
Source: Vaccines (Basel). 2021 Dec 23;10(1):14. doi: 10.3390/vaccines10010014 (PMC8778924; doi:10.3390/vaccines10010014)
Supplement: Supplementary file 1 [file vaccines-10-00014-s001.zip › vaccines-1478842-supplementary.pdf]

Supplementary Figure S1

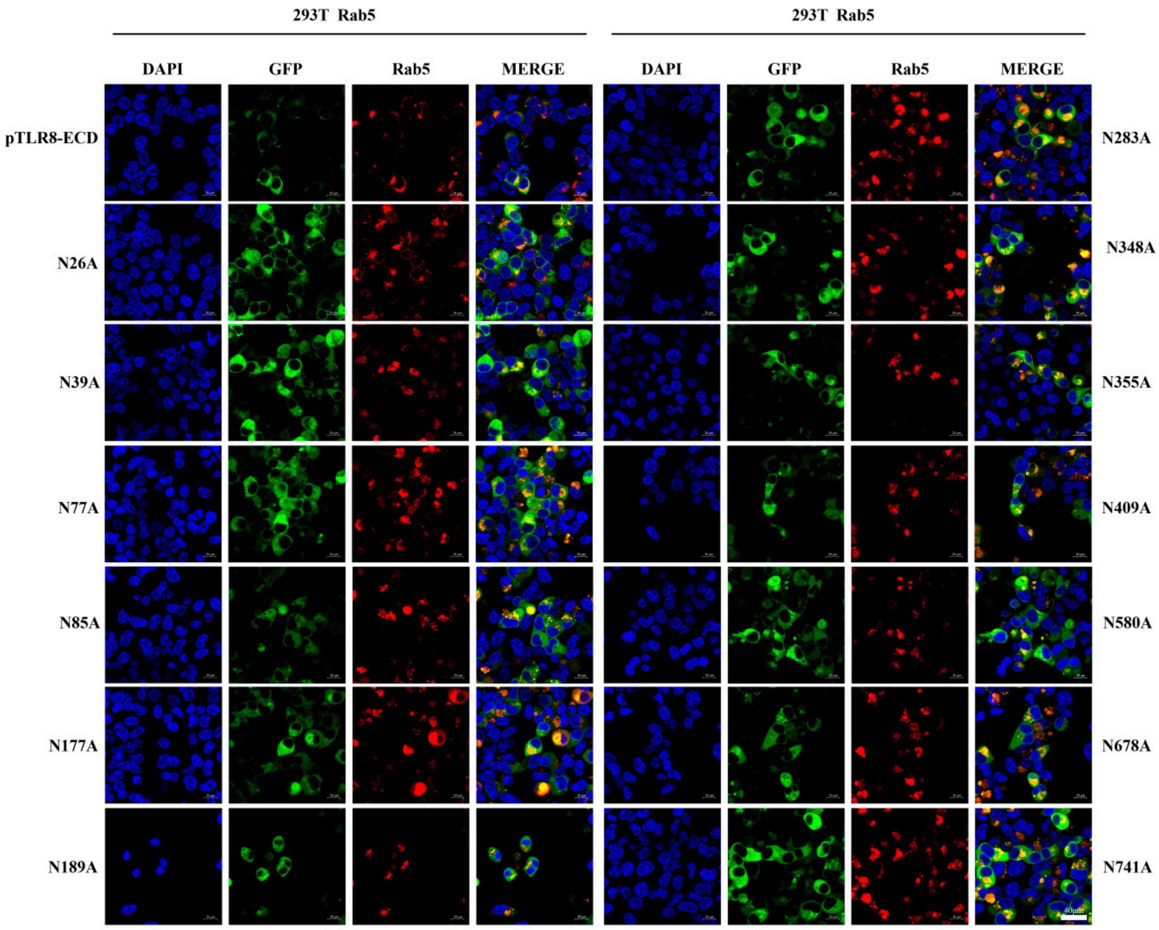

Supplementary Figure S1. The effect of N-glycosylation modification on the subcellular localization of pTLR8-ECD. HEK-293T cells (3×10^5 cells/well) were co-transfected with pDsRed-C1-pRab5 (0.5 μg) and pTLR8-ECD plasmids (0.5 μg) or the N-glycosylation site mutants of pTLR8-ECD plasmids (0.5 μg each) in confocal dishes. After DAPI staining, the cells were visualized under a confocal microscope. The scale bar is 40 μM.

Supplementary Figure S2

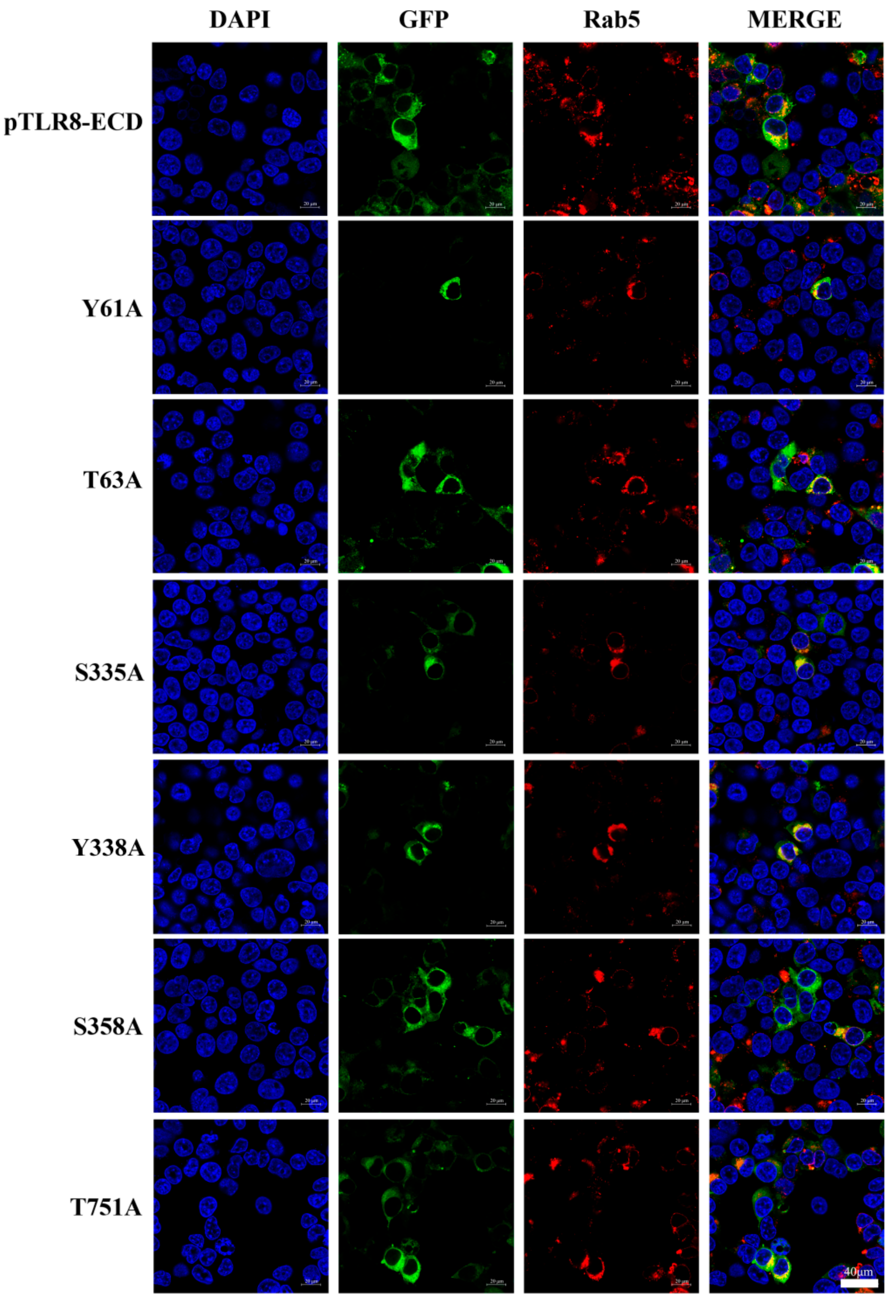

Supplementary Figure S2. The effect of phosphorylated modification on the subcellular localization of pTLR8-ECD. HEK-293T cells (3×10^5 cells/well) were co-transfected with pDsRed-C1-pRab5 (0.5 μg) and pTLR8-ECD plasmids (0.5 μg) or the phosphorylated modification site mutants of TLR8-ECD plasmids (0.5 μg each) in confocal dishes. After DAPI staining, the cells were visualized under a confocal microscope.

Supplementary Figure S3

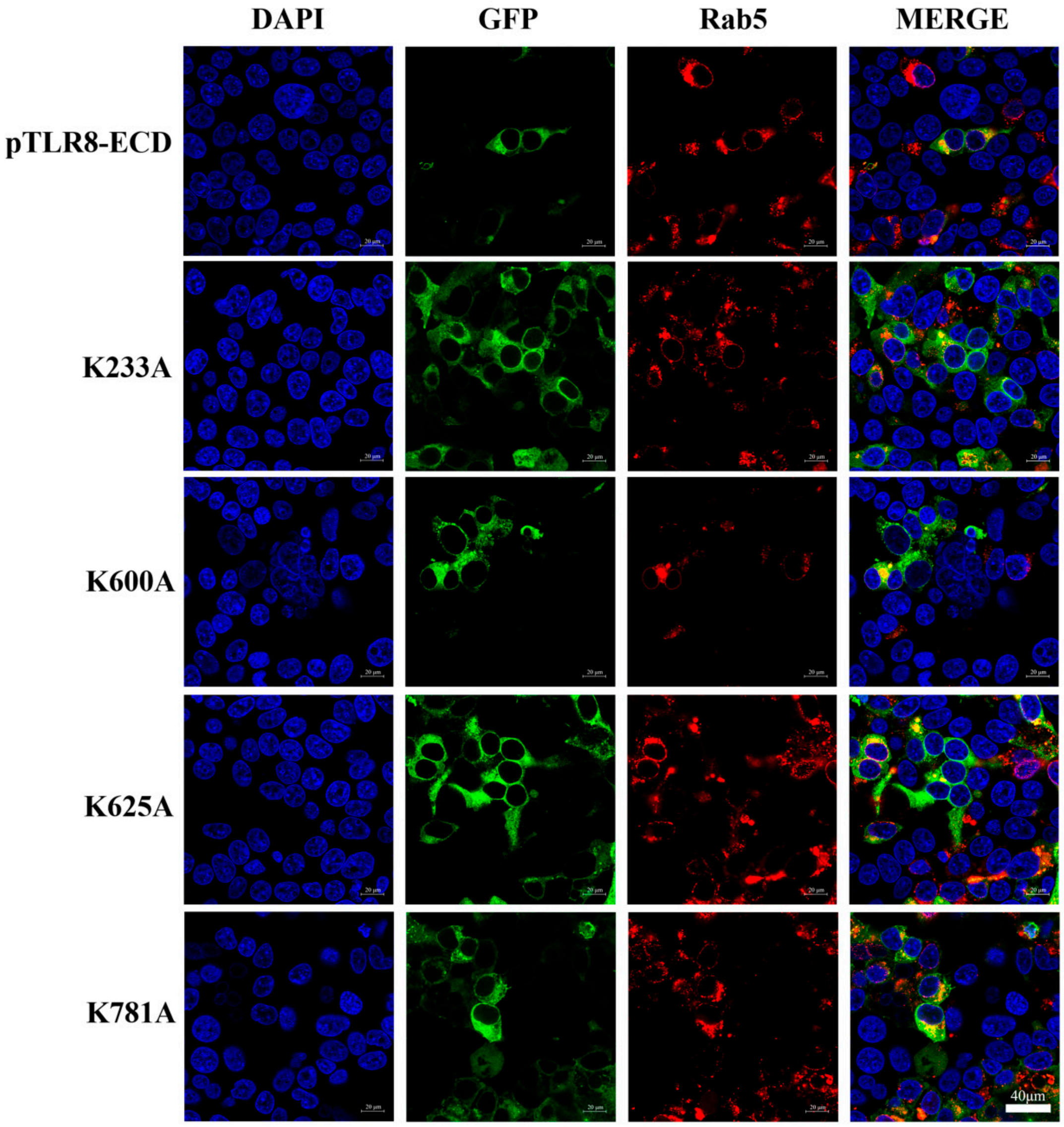

Supplementary Figure S3. The effect of ubiquitination modification on the subcellular localization of pTLR8-ECD. HEK-293T cells (3×10^5 cells/well) were co-transfected with pDsRed-C1-pRab5 (0.5 μg) and pTLR8-ECD plasmids (0.5 μg) or the ubiquitination modification site mutants of TLR8-ECD plasmids (0.5 μg each) in confocal dishes. After DAPI staining, the cells were visualized under a confocal microscope.

Supplementary Figure S4

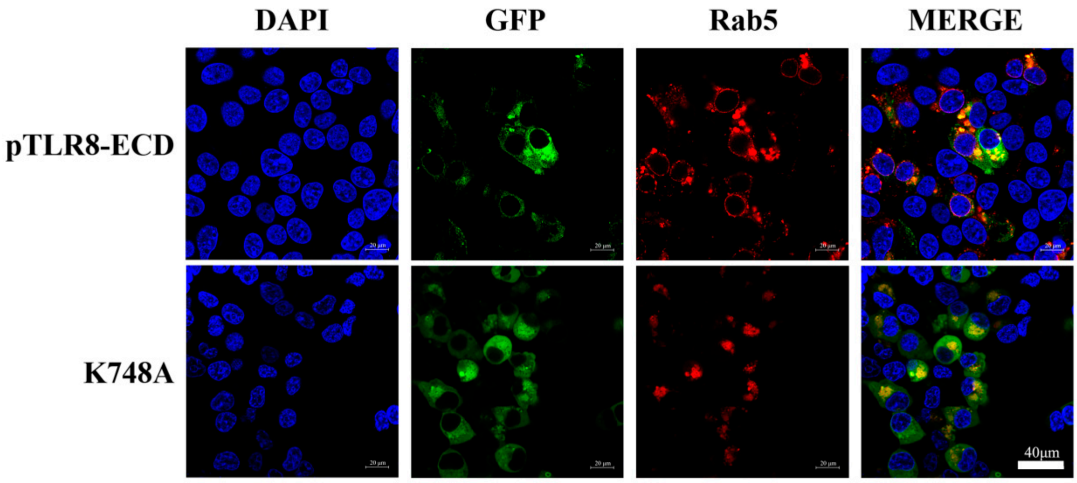

Supplementary Figure S4. The effect of acetylation modification on the subcellular localization of pTLR8-ECD. HEK-293T cells (3×105 cells/well) were co-transfected with pDsRed-C1-pRab5 (0.5 μg) and pTLR8-ECD plasmids (0.5 μg) or the acetylation modification site mutant of TLR8-ECD plasmids (0.5 μg) in confocal dishes. After DAPI staining, the cells were visualized under a confocal microscope.

Supplementary Figure S5

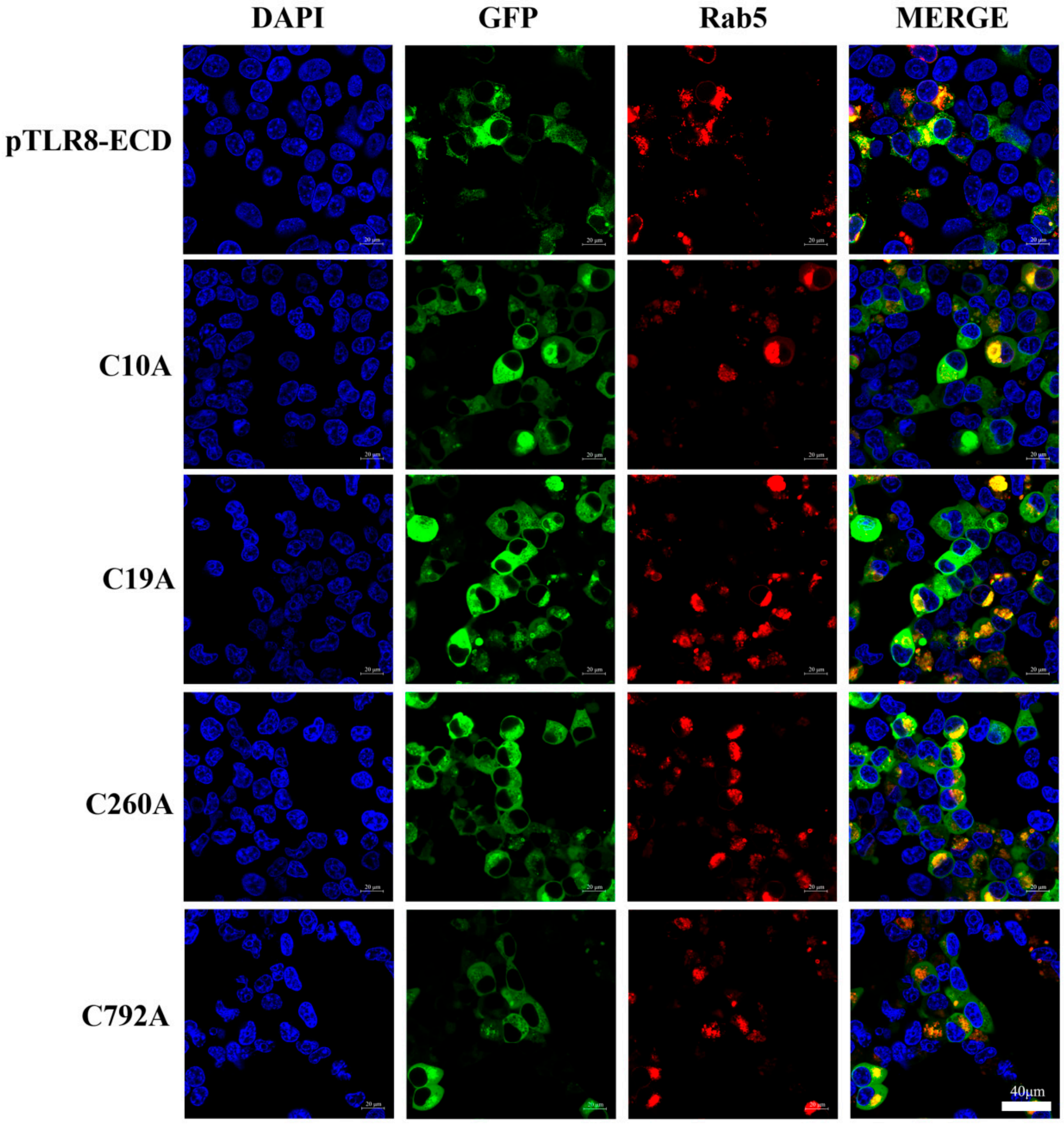

Supplementary Figure S5. The effect of palmitoylation modification on the subcellular localization of pTLR8-ECD. HEK-293T cells (3×10^5 cells/well) were co-transfected with pDsRed-C1-pRab5 (0.5 μg) and pTLR8-ECD plasmids (0.5 μg) or the palmitoylation modification site mutants of TLR8-ECD plasmids (0.5 μg each) in confocal dishes. After DAPI staining, the cells were visualized placed under a confocal microscope.

Supplementary Figure S6

Figure 1A

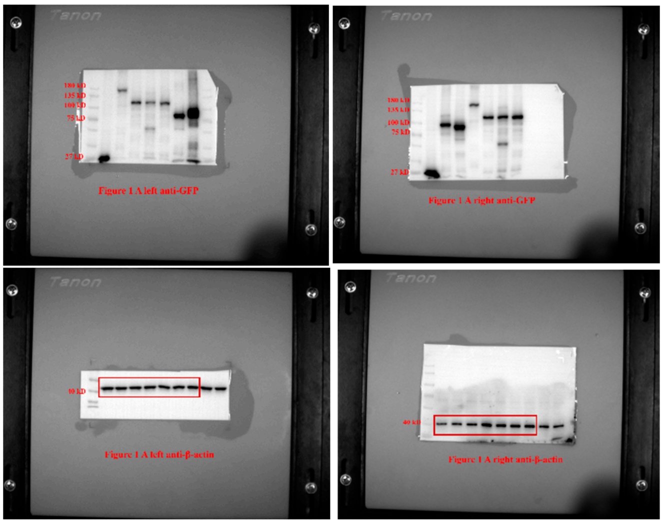

Figure 1B

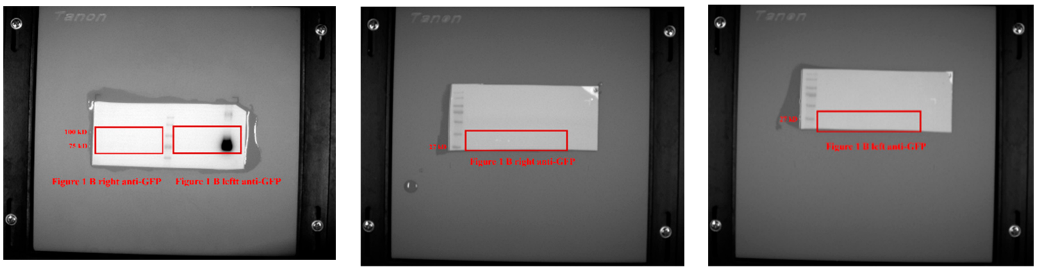

Figure 4A

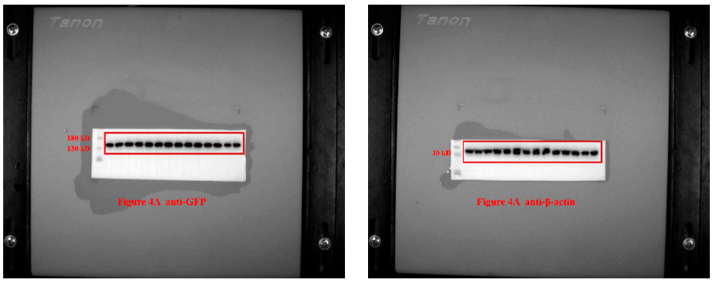

Figure 4B

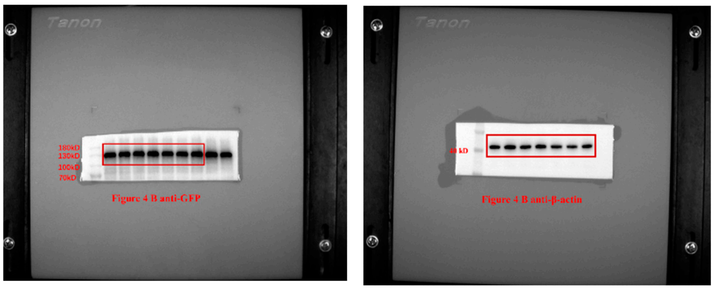

Figure 4C

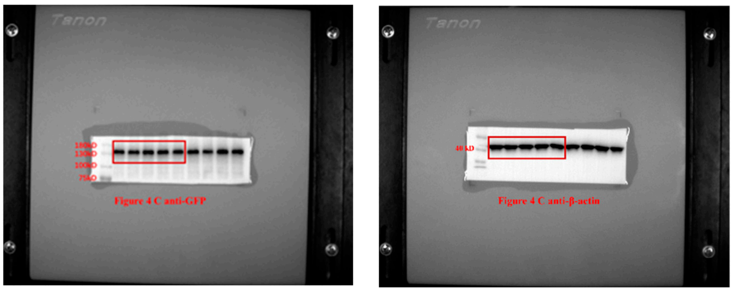

Figure 4D

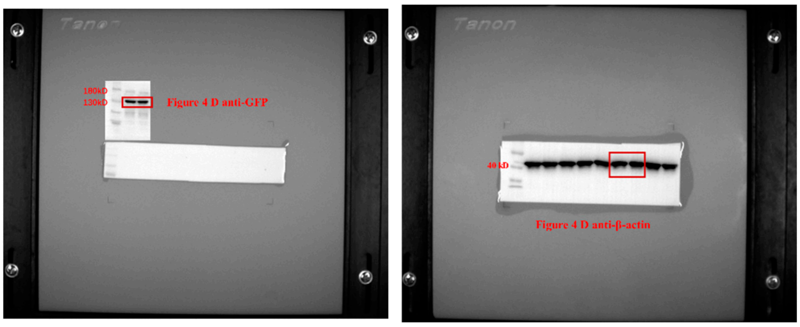

Figure 4E

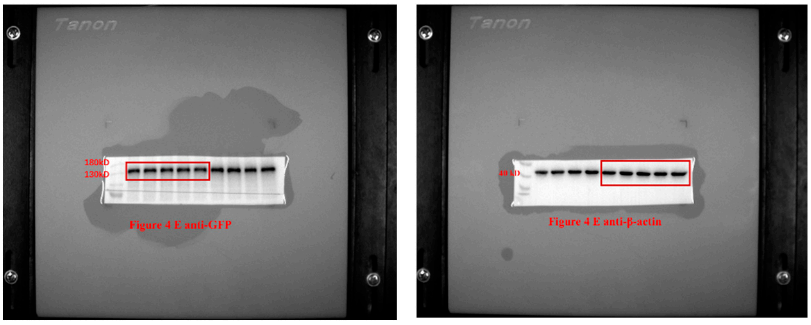

Figure 5A

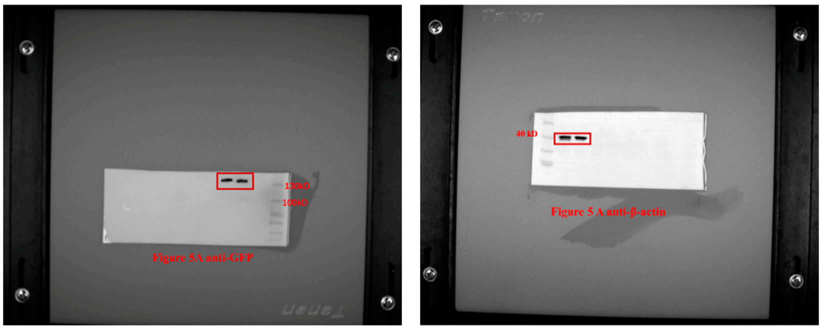

Figure 5C

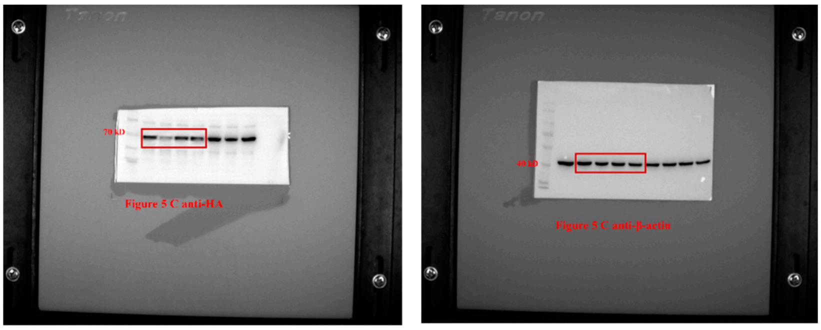

Supplementary Figure S6. The whole blot (uncropped blots) showing all the bands with all molecular weight markers on the blots.

Supplementary Table S1. Cloning PCR Primers used in this study

Vectors	Target Genes	Primer Sequences
pEGFP-N1	hTLR8-ECD	5'-agcgctaccggactcagatctatggaaaacatgttccttcag-3'<br>5'-ggatcccgggcccgcggtaccgtatctgaaacacaagttgttag-3'
pEGFP-N1	bTLR8-ECD	5'-agcgctaccggactcagatctatgacccttcactttttgctt-3'<br>5'-ggatcccgggcccgcggtaccgtatctgaaacacaagtgctgag-3'
pEGFP-N1	pTLR8-ECD	5'-agcgctaccggactcagatctatgacccttcactttttgctc-3'<br>5'-ggatcccgggcccgcggtaccgtggtatccgaaacacaagtt-3'
pEGFP-N1	pTLR8	5'-agcgctaccggactcagatctatgacccttcactttttgctc-3'<br>5'-ggatcccgggcccgcggtaccgtcttaatggaattgacata-3'
pEGFP-N1	gD	5'-agcgctaccggactcagatctatgcaagggccgacattggcc-3'<br>5'-ggatcccgggcccgcggtaccgtcccgggcagcgcgctgtagtt-3'
pEGFP-N1	gD-ECD	5'-agcgctaccggactcagatctatgcaagggccgacattggcc-3'<br>5'-ggatcccgggcccgcggtaccgtgtcgggggccgcgggcgtagc-3'
pEGFP-C1	hTLR8-ECD	5'-tacaagtccggactcagatctatggaaaacatgttccttcag-3'<br>5'-ggatcccgggcccgcggtaccttaatctgaaacacaagttgttag-3'
pEGFP-C1	bTLR8-ECD	5'-tacaagtccggactcagatctatgacccttcactttttgctt-3'<br>5'-ggatcccgggcccgcggtaccttaaccgtatctgaaacacaagtgctgag-3'
pEGFP-C1	pTLR8-ECD	5'-tacaagtccggactcagatctatgacccttcactttttgctc-3'<br>5'-ggatcccgggcccgcggtaccttaaccgtggtatccgaaacacaagtt-3'
pEGFP-C1	pTLR8	5'-tacaagtccggactcagatctatgacccttcactttttgctc-3'<br>5'-ggatcccgggcccgcggtaccttacttaatggaattgacataca-3'
pEGFP-C1	pTLR5-ECD	5'-tacaagtccggactcagatctatgggagactgcctggtcctg-3'<br>5'-ggatcccgggcccgcggtaccttaccggagatccaaggtatttaa-3'
pEGFP-C1	pTLR3-ECD	5'-tacaagtccggactcagatctatgagcaggagtttgccttgt-3'<br>5'-ggatcccgggcccgcggtaccttaattaatccaactaacaaacca-3'
pDsRed-C1	hGM130	5'-gaagatctatgtcggaagaaacccgacag-3'<br>5'-ggggtaccgatgacagtgatcttcacctc-3'
pDsRed-C1	hLAMP1	5'-gaagatctatggcgccccgcagcgcccgg-3'<br>5'-ggggtaccgatagtctggtagcctgcgtga-3'
pDsRed-C1	pSTING	5'-gaagatctatgccctactccagcctgcat-3'

		5'-ggggtacctcagaagatatctgagcggag-3'
pcDNA3.1-2HA	pUNC93B1	5'-gggagacccaagctggctagcgccaccatggaggcggagccgccgct-3'
		5'-gtatgggtagctggtgatatcctgctcctccggcccatcgcc-3'

Note: the restriction sites are underlined.

Supplementary Table S2. Mutation PCR primers used for pTLR8-ECD mutants

Vectors	Mutation Sites	Primer Sequences
pEGFP-C1	N26A	5'-aagggtagcttctagaataagcggctccagtgaagaactcac-3'
		5'-gtgagttcttcactggagccgcttattctagaagctaccctt-3'
pEGFP-C1	N39A	5'-caatgacagagccagcttcttttctctcatcacaagggtagct-3'
		5'-agctacccttgtgatgagagaaaagaagctggctctgtcattg-3'
pEGFP-C1	N77A	5'-cagcccttgaaaggattcagcggttatgcgtctgatgaag-3'
		5'-cttcatcagacgcataaccgctgaatcctttcaagggctg-3'
pEGFP-C1	N85A	5'-tggtttagatttattttagtcagagcttgcagcccttgaaaggattcattgg-3'
		5'-ccaatgaatcctttcaagggctgcaagctctgactaaaataaatctaaacca-3'
pEGFP-C1	N177A	5'-cgtctatgataaaggtttcagcacaagtaaaatagcagttccagcccaaata-3'
		5'-tatttgggctggaactgctattttacttgtgctgaaacctttatcatagacg-3'
pEGFP-C1	N189A	5'-cagcaccttcaaattcgttaaagcttcaaatgctccctcgtctatg-3'
		5'-catagacgagggagcatttgaagctttaacgaatttgaaggtgctg-3'
pEGFP-C1	N283A	5'-cgaagtgctagagagggctaggtagcgaagttcggtc-3'
		5'-gaccgaacttcgctacctagccctctctagcacttcg-3'
pEGFP-C1	N348A	5'-gtaagattagcaaagtaatgggaaatagcaatgtactggggatattttttctgttc-3'
		5'-gaacagaaaaaatatccccagtacattgctatttcccattactttgctaatcttac-3'
pEGFP-C1	N355A	5'-gtgtaatatctggagagatgtaagagcagcaaagtaatgggaaatattaatgtact-3'
		5'-agtacattaatatttcccattactttgctgctcttacatctctccagatattacac-3'
pEGFP-C1	N409A	5'-gacaagtaaatgattgacagggcggagaactccgagaaaatggt-3'
		5'-accattttctcggagttctccgccctgtcaatcatttacttgtc-3'
pEGFP-C1	N580A	5'-ctgttgtggctcaaggctaaaactctcagctgaggcaaattttg-3'
		5'-caaaatttgcctcagctgagagttttagccttgagccacaacag-3'
pEGFP-C1	N678A	5'-ttaagtcaagcaaggtgagagccggaaactgctggagtaatg-3'

		5'-cattactccagcagtttccggctctcaccttgcttgacttaa-3'
pEGFP-C1	N741A	5'-tgcgtgaagcttggatatggcgaccatcttgagctggttg-3'<br>5'-caaccagctcaagatggtcgccatatccaagcttcacgca-3'
pEGFP-C1	Y61A	5'-agtccagttcagtcacagcgttgcccactcttcggg-3'<br>5'-cccgaagagtgggcaacgctgtgactgaactggact-3'
pEGFP-C1	T63A	5'-agacaagtccagttcagccacatagttgcccactc-3'<br>5'-gagtgggcaactatgtggctgaactggacttgtct-3'
pEGFP-C1	S335A	5'-attttttctgttcatagttgtaagctaagtcaagtatttctaaggaggg-3'<br>5'-ccctccttagaaatacttgacttagcttacaactatgaacagaaaaaat-3'
pEGFP-C1	Y338A	5'-aatgtactggggatatttttctgttcagcgttgtaagataagtcaagtatttctaag-3'<br>5'-cttagaaatacttgacttatcttacaagctgaacagaaaaaatatccccagtacatt-3'
pEGFP-C1	S358A	5'-ttaagtgtaatatctggagagctgtaagattagcaaagtaatggga-3'<br>5'-tcccattactttgctaatcttacagctctccagatattacacttaa-3'
pEGFP-C1	T751A	5'-ttaaaatggctaagttggcggtggtctttgcgtgaag-3'<br>5'-cttcacgcaaagaccaccgccaacttagccattttaa-3'
pEGFP-C1	K748A	5'-ggctaagttggtggtggtcgctgcgtgaagcttggatatg-3'<br>5'-catatccaagcttcacgcagcgaccaccaccaacttagcc-3'
pEGFP-C1	K233A	5'-ctcttagattttccagccccgcgaagtcttcctgattgatgt-3'<br>5'-acatcaatcaggaagacttcgcggggctggaaaatctaagag-3'
pEGFP-C1	K600A	5'-ggtttccactgaaaactaattctgccagggacgtgctttttaagtacg-3'<br>5'-cgtacttaaaaagcacgtccctggcagaattagttttcagtggaaacc-3'
pEGFP-C1	K625A	5'-ggtcagcgtgctgagatttgcgaaaatttgccagtacctg-3'<br>5'-caggtactggcaaattttcgcaaatctcagcacgctgacc-3'
pEGFP-C1	K781A	5'-cagtctgggaattgtgaccgccagattctcatccatccat-3'<br>5'-atggatggatgagaatctggcggtcacaattcccagactg-3'
pEGFP-C1	C10A	5'-ggaattcgcaggaaaagggcggtcaggagcaaaaagtg-3'<br>5'-cactttttgctcctgaccgcccttttcctgcgaattcc-3'
pEGFP-C1	C19A	5'-gctccagtgaagaactcagcggaatcaggaattcgcag-3'<br>5'-ctgcgaattcctgattccgctgagttcttcactggagc-3'
pEGFP-C1	C260A	5'-atctccgggggcagggttgcagggaaagggt-3'<br>5'-accctttccctgcaaccctgcccccggagat-3'

pEGFP-C1	C792A	5'-caggactggcagcaatgacatccgtcagtctggg-3'<br>5'-cccagactgacggatgtcattgctgccagtcctg-3'
pEGFP-C1	Δ-SP	5'-tacagtccggactcagatctgagttcttcactggagccaat-3'<br>5'-ggatcccgggcccgcggtaccttaaccgtggtatccgaaacacaagtt-3'

Supplementary Table S3. Specific guide RNA coding sequences for porcine UNC93B1 CRISPR knockout

Target Gene	gRNAs	Primer Sequences
UNC93B1	gRNA-1	5'-CACCGcccgccacctggtagagcgg-3'<br>5'-aaacccgctctaccaggtggcgggC-3'
	gRNA-2	5'-CACCGggcggagccgccgctctacc-3'<br>5'-aaacggtagagcggcggctccgcc-3'
	gRNA-3	5'-CACCGctaccaggtggcgggagccg-3'<br>5'-aaaccggctcccgccacctggtagC-3'
